# Chromosome level genome assembly of ‘Wanfeng’ almond (*Prunus dulcis*)

**DOI:** 10.1038/s41597-025-04480-4

**Published:** 2025-01-30

**Authors:** Dongdong Zhang, Zhenfan Yu, Xingyue Liu, Yong Li, Bin Zeng, Lirong Wang

**Affiliations:** 1 College of Horticulture, Xinjiang Agricultural University, Urumqi, 830000 China; 2 Western Research Institute, CAAS, Changji, Xinjiang 831100 China

**Keywords:** Genomics, Plant sciences

## Abstract

We assembled a chromosome-level genome of Chinese native ‘Wanfeng’ almond, with a size of 288.53 Mb and a contig N50 of 30.48 Mb. Approximately 270 Mb (93.58%) of the sequences are anchored on 8 Superscaffolds, and 174.59 Mb (60.51%) of the sequences are repetitive sequences. BUSCO assessment revealed that the ‘Wanfeng’ almond genome assembly included 99.3% complete BUSCOs. A total of 24,230 protein-coding genes were annotated, and 24,033 were functional. The assembly of the ‘Wanfeng’ almond genome provides a valuable genetic resource for molecular breeding of native almonds in China.

## Background & Summary

*Prunus dulcis* (Mill.) D. A. Webb is a fruit tree of the genus *Amygdalus* in the Rosaceae family. Almond is an important economic fruit tree that is widely planted worldwide and is one of the four largest dried fruits in the world^1^. Almond have been cultivated in China since the Tang Dynasty, with a history of more than 1,300 years. The Kashgar region in Xinjiang, China, is the main production area for cultivated almond plants and has abundant almond resources, including ‘Duoguo’, ‘Shuangruan’, ‘Wanfeng’, ‘Zhipi’, ‘Yingzui’ and ‘Shuangguo’. To date, relevant research institutions in Europe and the United States have sequenced and assembled the genomes of three almond varieties, ‘Lauranne’^2^, ‘Texas’^3^ and ‘Nonpareil’^4^, and have achieved important results. Therefore, it is imperative to conduct genome sequencing and assembly for native almond varieties in China.

**Fig. 1:**
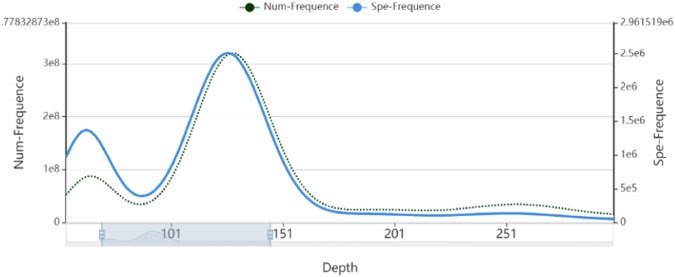
*K*-mer depth and *K*-mer species number frequency distribution plot. *K*-mer = 17. *K*-mer depth = 126.

**Fig. 2:**
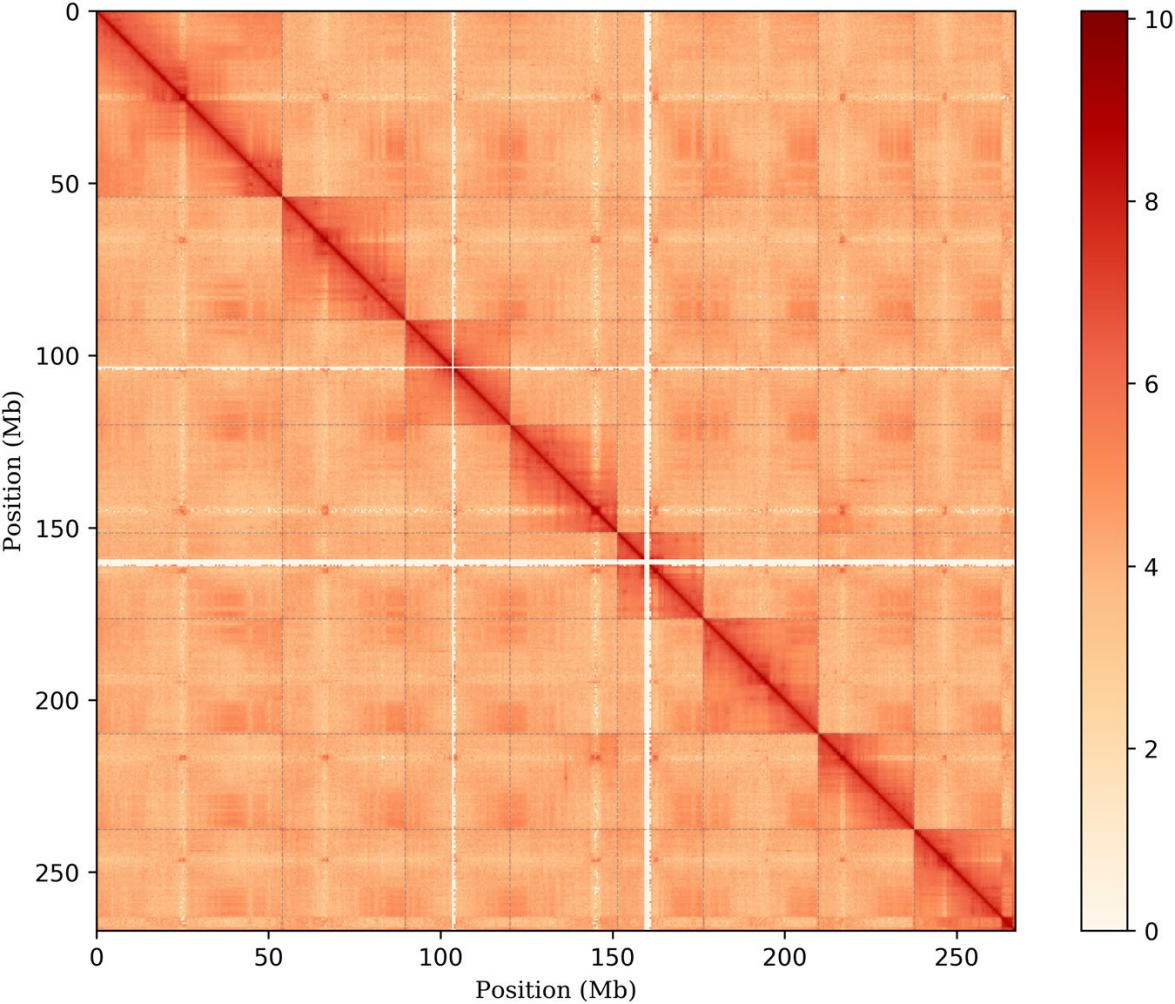
Hi-C interactive heatmap of ‘Wanfeng’ almond. The color in the figure from light to dark indicates the increase in the interaction strength, and the darker the color is, the stronger the interaction.

**Fig. 3:**
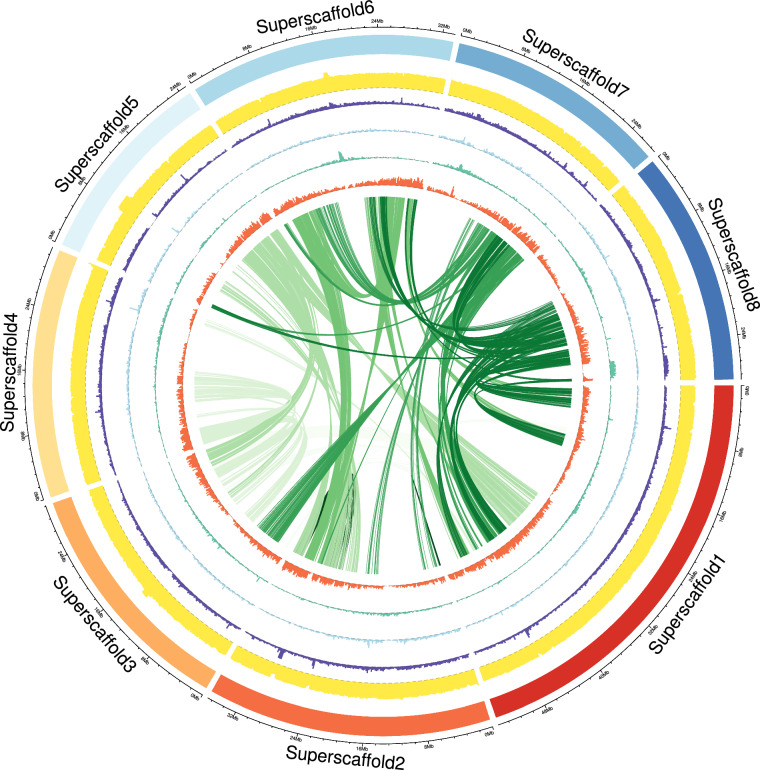
Genome map of ‘Wanfeng’ almond. The circle represents the distribution of chromosomes, GC density, LTR density, *Copia* LTR transposon density, and *Gypsy* LTR transposon density of ‘Wanfeng’ almond from the outside to the inside, in a 200 kb non overlapping window. The genomic collinearity between homologous chromosomes is located in the center. Superstffold represents chromosome.

We sequenced the ‘Wanfeng’ almond leaves using the DNBSEQ-T7 platform and obtained 50.29 Gb of second-generation raw data. *K*-mer analysis predicted the genome size of ‘Wanfeng’ almond to be 288.08 Mb, with a heterozygosity of 0.81% and a repeat sequence proportion of 50.98% (Fig. 1, Table 1). Subsequently, HiFi (High-Fidelity) data was obtained through the PacBio Sequel II sequencing platform to assemble the ‘Wanfeng’ almond *de novo* genome, and was combined with Hi-C sequencing data to assist in the assembly and reach the chromosome level (Fig. 2, Table 1). Finally, the genome size of ‘Wanfeng’ almond was 288.53 Mb, the contig N50 and scaffold N50 were 30.48 Mb and 31.36 Mb respectively, and approximately 270 Mb of sequence were anchored on 8 Superscaffolds. We predicted a total of 24,230 protein-coding genes in the ‘Wanfeng’ almond genome and performed functional and structural domain annotations in five databases: InterPro^5^, Gene Ontology (GO)^6^, Kyoto Encyclopedia of genes and Genomes (KEGG)^7^, SwissProt, and NCBI Non-redundant protein (NR)^8,9^ (Table 2). In addition, 225 miRNAs, 530 tRNAs, 7669 rRNAs, and 816 snRNAs non-coding RNA were also identified (Table 3). About 60.51% of repetitive sequences were detected in the genome, LTR (Long terminal repeat) transposons made up 44.22% of the total genome, while DNA transposons accounted for 11.44% (Table 4). The ‘Wanfeng’ almond genome can fully map 1599 Benchmarking Universal Single-Copy Orthologs (BUSCO) genes^10^, accounting for 99.3% (Table 5). This study obtained a high-quality chromosome level genome of ‘Wanfeng’ almond (Fig. 3).

**Table 1:** Statistics of the ‘Wanfeng’ almond genome assembly and annotation.

ASSEMBLY FEATURE	STATISTIC
**RAW DATA OF DNBSEQ-T7 SEQUENCING (GB)**	50.29
**RAW DATA OF PACBIOO-CCS SEQUENCING (GB)**	402.27
**RAW DATA OF HI-C SEQUENCING (GB)**	41.15
**FILTERED HIFI DATA (GB)**	27.57
**ESTIMATED GENOME SIZE (DNBSEQ-T7 SEQUENCING READS: MB)**	288.08
**ESTIMATED GENOME SIZE (HIFI SEQUENCING READS: MB)**	316.25
**ASSEMBLED GENOME SIZE (MB)**	288.53
**CONTIG N50 (MB)**	30.48
**NUMBER OF CONTIG**	213
**LARGEST CONTIG (MB)**	1.29
**TOTAL SIZE OF CHROMOSOME (MB)**	267.49
**GC CONTENT (%)**	39.10
**HETEROZYGOSITY (%)**	0.81
**NUMBER OF GENES**	24,230
**BUSCO SCORES (%)**	99.30
**HOMOLOGY INDEL**	4,319

**Table 2:** Statistics of function comments.

Types	Gene numbers	Percent (%)
InterPro	18,342	75.70
GO	19,314	79.71
KEGG	23,241	95.92
Swissprot	17,194	70.96
TrEMBL	24,000	99.05
NR	24,025	99.15
Annotated	24,033	99.19
Unannotated	197	0.81
Total	24,230	100

**Table 3:** Statistics of non-coding RNA annotation results of ‘Wanfeng’ almond genome.

Types		Copy	Average length (bp)	Total length (bp)	Percent (%)
miRNA		225	117.7511111	26,494	0.009182
tRNA		530	76.63207547	40,615	0.014077
rRNA	rRNA	7,669	2236.374886	17,150,759	5.944196
	18S	2,173	1803.043258	3,918,013	1.357925
	28S	2,170	5921.832258	12,850,376	4.453748
	5S	3,326	114.9639206	382,370	0.132524
snRNA	snRNA	816	111.7303922	91,172	0.031599
	CD-box	664	104.7319277	69,542	0.024102
	HACA-box	36	128.9722222	4,643	0.001609
	splicing	116	146.4396552	16,987	0.005887
	scaRNA	0	0	0	0

**Table 4:** Repeat sequence classification statistics.

Types	Repeat Masker TEs Length (bp)	Repeat Masker TEs % in genome	Repeat Protein Mask TEs Length (bp)	Repeat Protein Mask TEs % in genome	*De novo* Length (bp)	*De novo* % in genome	Combined TEs Length (bp)	Combined TEs % in genome
DNA	18,189,485	6.3	3,280,184	1.14	20,284,156	7.03	33,005,297	11.44
LINE	2,008,545	0.7	487,137	0.17	2,002,978	0.69	3,639,410	1.26
SINE	27,960	0.01	0	0	0	0	27,960	0.01
LTR	36,978,199	12.82	18,401,430	6.38	119,351,329	41.37	127,594,571	44.22
Other	304	0	0	0	0	0	304	0
Unknown	181,560	0.06	0	0	20,250,051	7.02	20,418,496	7.08
Total TE	56,239,181	19.49	22,168,099	7.68	154,627,003	53.59	171,772,469	59.53

**Table 5:** BUSCO assesses the integrity of the ‘Wanfeng’ almond genome assembly.

Term	BUSCO number	Proportion (%)
Complete BUSCOs	1,599	99.3
Complete and single-copy BUSCOs	1,568	97.1
Complete and duplicated BUSCOs	31	1.9
Fragmented BUSCOs	3	0.2
Missing BUSCOs	12	0.8
Total BUSCO groups searched	1,614	100

## Methods

### Sample collection and genome size estimation

We selected a robust six-year-old ‘Wanfeng’ almond plant from the state-owned Erlinchang Almond Resource Garden in Yarkand County, Kashgar Prefecture, Xinjiang Uygur Autonomous Region, for genome sequencing and *de novo* assembly. Fresh intact leaves were collected from the ‘Wanfeng’ almond plant for extraction of genomic DNA, and young leaves were extracted for full-length transcriptome sequencing for genome-assisted annotation. The collected tissues were immediately stored at −80 °C until further use.

The full-length cDNA was prepared using a SMARTer^TM^ PCR cDNA Synthesis Kit (Takara Biotechnology, Dalian, China). The SMRTbell libraries were constructed with the Pacific Biosciences DNA Template Prep Kit 2.0. Library. Library quantification and size were measured using a Qubit 3.0 Fluorometer (Life Technologies, Carlsbad, CA, USA) and a Bioanalyzer 2100 system (Agilent Technologies, CA, USA). Subsequently, SMRT sequencing was performed on a PacBio Sequel II platform by Frasergen Bioinformatics Co., Ltd. (Wuhan, China).

The qualified ‘Wanfeng’ almond leaves DNA samples were fragmented by ultrasonic disruption to lengths of 300 to 500 bp. Subsequently, the samples underwent end repair, A-tailing, sequencing adapter ligation, purification, PCR amplification, and other steps to complete the library preparation. The sequencing data generation involved multiple steps including DNA extraction, library construction, and sequencing. We used SOAPnuke software^11^ to filter the raw reads, resulting in high-quality reads, with main parameters set as: --lowQual = 20,--nRate=0.005, --qualRate=0.5, and other parameters were set to default. We employed a *K*-mer-based^12^ analysis method to estimate the genome size, heterozygosity rate, and repetitive sequence information of the ‘Wanfeng’ almond genome.

### HiFi and Hi-C library construction and sequencing

We used g-TUBE (Covaris) to randomly fragment the extracted ‘Wanfeng’ almond leaves DNA into approximately 15 kb fragments. The SMRTbell Express Template Prep Kit 2.0 (Pacific Biosciences) was then utilized to construct the SMRT bell HiFi library. Raw data obtained from sequencing on the PacBio Sequel II platform were processed using SequelQC software^13^ to remove low-quality regions and adapter sequences.

The ‘Wanfeng’ almond leaves cell chromatin was treated with formaldehyde to fix the DNA conformation. The cross-linked DNA was then digested with restriction enzymes, while simultaneously introducing biotin to label the oligonucleotide ends. The DNA fragments underwent end repair, A-tailing, adapter ligation, evaluation of PCR amplification cycles, and purification to complete the library preparation. After the Hi-C library construction^14,15^, different libraries were pooled based on their effective concentrations and the desired amount of data for sequencing on the BGI MGISEQ-2000 PE150 (Paired-end 150 bp) platform.

### ***De novo*** genome assembly

SMRTbell libraries were sequenced on a PacBio Sequel II systemand consensus reads (HiFi reads) were generated using ccs software (https://github.com/pacificbiosciences/unanimity) with the parameter ‘-minPasses 3’. To further validate and improve the assemblies, we generated 37–81 × PacBio HiFi reads for the three accessions. These long (~15 kb) and highly accurate (>99%) HiFi reads were assembled using hifiasm (Version: 0.14-r312)^16^ with default parameters, and the GFAtools (https://github.com/lh3/gfatools)^17^ was used to convert sequence graphs in the GFA to FASTA format.

For anchored contigs, 141,649,186 clean reads were generated from the Hi-C library and were mapped to the ‘Wanfeng’ almond preliminary assembly using Juicer^14^ with default parameters. Paired reads mapped to different contigs were used for the Hi-C associated scaffolding. Self-ligated, non-ligated, and other invalid reads were filtered out. We applied 3D-DNA^18^ to order and orient the clustered contigs. Then, Juicer was used to filter the sequences and cluster them, and the Juicebox was applied to adjust chromosome construction manually. We finally anchored the scaffolds on 8 chromosomes. In addition, the BUSCO pipeline was used to assess the completeness and accuracy of the ‘Wanfeng’ almond genomes.

### Annotation of repetitive sequences

The two methods were combined to identify the repeat contents in our genome via homology-based and *de novo* prediction. Homology-based analysis: We identified known transposable elements (TEs) within the ‘Wanfeng’ almond genome using RepeatMasker (Version: open-4.0.9) with the Repbase TE library^19–21^. RepeatProteinMask searches were also conducted using the TE protein database as a query library. *De novo* prediction: We constructed a *de novo* repeat library of the ‘Wanfeng’ almond genome using RepeatModeler (http://www.repeatmasker.org/RepeatModeler/), which can automatically execute two core *de novo* repeat-finding programs, namely, RECON (Version: 1.08) and RepeatScout (Version: 1.0.5), to comprehensively construct, refine and classify consensus models of putative interspersed repeats for the ‘Wanfeng’ almond genome^22,23^. Furthermore, we performed a *de novo* search for LTR retrotransposons against the ‘Wanfeng’ almond genome sequence using LTR_FINDER (Version: 1.0.7)^24^. We also identified tandem repeats using the Tandem Repeat Finder (TRF)^25^ package and noninterspersed repeat sequences, including low-complexity repeats, satellites and simple repeats, using RepeatMasker. Finally, we merged the library files of the two methods and used a repeatmaker to identify the repeat contents.

### Annotation of protein-coding genes

We predicted protein-coding genes in the ‘Wanfeng’ almond genome using three methods, namely, ab initio gene prediction, homology-based gene prediction and full-length transcriptome-aided gene prediction. Prior to gene prediction, the assembled ‘Wanfeng’ almond genome was hard and soft masked using RepeatMasker. We selected Augustus (Version: 3.3.1) and Genescan for ab initio gene prediction^26–29^, 2000 correct genes predicted by homology-based gene prediction were used as training gene set. Models used for each gene predictor were trained from a set of high-quality proteins generated from the full-length transcriptome dataset. We used Exonerate (Version: 2.2.0) to conduct homology-based gene prediction^30^. First, the protein sequences were aligned to our genome assembly, and the coding genes were predicted using Exonerate with the default parameters. For full-length transcriptome-based gene prediction, the reads of the full-length transcriptome were aligned to scaffolds using GMAP^31^. The transcripts were used to predict open reading frames (ORFs) using PASA (Version: 2.0.1) (https://sourceforge.net/projects/pasa/files/stats/timeline), and full-length cDNA was screened as a training set^32^. Finally, Maker (Version: 3.00) was used to integrate the prediction results of the three methods to predict gene models^33^. The output included a set of consistent and nonoverlapping sequence assemblies, which were used to describe the gene structures.

### Functional annotation of protein-coding genes

Gene functions were inferred according to the best match of the alignments to the National Center for Biotechnology Information (NCBI) Nonredundant, InterPro and Swiss-Prot protein databases using BLASTP (Version: 2.6.0)^34^ and the KEGG database with an E-value threshold of 1E^−5^. The protein domains were annotated using PfamScan (pfamscan_version) and InterProScan (Version: 5.35–74.0) based on InterPro protein databases^35,36^. The motifs and domains within the gene models were identified via the PFAM database. Gene Ontology (GO) IDs for each gene were obtained from Blast2GO^37^. In total, approximately 24,033 (approximately 99.19%) of the predicted protein-coding genes of ‘Wanfeng’ almond could be functionally annotated with known genes, conserved domains, and GO terms (Table 2).

### Annotation of Non-coding RNA genes

We used the tRNAscan-SE (Version: 1.3.1) algorithm with default parameters to identify the genes associated with tRNA^38^. For rRNA identification, we first downloaded rRNA sequences from closely related species from the Ensembl database. Then, the rRNAs in the database were aligned again our genome using BLASTN with a cutoff of E-value < 1e^−5^, identity ≥85% and match length ≥50 bp. MiRNAs and snRNAs were identified by Infernal (Version: 1.1.2) software against the Rfam (Version: 14.1) database with default parameters^39,40^.

### Genome quality evaluation

To examine the assembly integrity, the HiFi reads were aligned again onto the final assembly using minimap2 (Version: 2.5) with default parameters^41^. A total of 100% of the raw reads were mapped. The assembled genomes were also subjected to BUSCO with OrthoDB to evaluate the completeness of the genome. Overall, 99.3% complete and 0.7% missing BUSCOs were identified in the assembled genome (Table 5). Short reads were aligned from the Illumina platform to the genome; thus, the high alignment ratio and single-peak insertion length distribution demonstrated the high quality of the contig assembly. To evaluate the accuracy of the genome at the single-base level, using Illumina short read alignment to the reference genome of the Wanfeng almond by BWA (Version: 0.7.17) software^42^, we identified 4,319 homology indel loci (0.0008% of the total ‘Wanfeng’ almond assembly) via the GATK (Version: 4.0.8.1) package (Table 1)^43^.

## Data Records

The DNBSEQ-T7 short reads, PacBio Sequel II long-reads, and Hi-C sequencing data used for the genome assembly have been deposited in the China National Center for Bioinformation Genome Sequence Archive (GSA) database with the GSA number CRA016115^44^. The whole genome sequence data reported in this paper have been deposited in the Genome Warehouse in National Genomics Data Center, Beijing Institute of Genomics, Chinese Academy of Sciences/China National Center for Bioinformation, under accession number GWHERKB00000000 that is publicly accessible at https://ngdc.cncb.ac.cn/gwh/Assembly/83927/show^45^. The genome annotation files are available in Figshare (10.6084/m9.figshare.27917241.v1)^46^. This Whole Genome Shotgun project has been deposited at DDBJ/ENA/GenBank under the accession JBELOH000000000. The version described in this paper is version JBELOH010000000^47^. The transcriptome and full-length transcriptome (Iso-Seq) data used to assist in the annotation of the ‘Wanfeng’ almond genome have been deposited in the Genome Sequence Archive (GSA) database of the China National Center for Bioinformation under the GSA accession number CRA020887^48^.

## Technical Validation

Firstly, we sequenced the ‘Wanfeng’ almond genome on the DNBSEQ-T7 platform, generating 50.29 Gb of raw data for genome survey analysis. Through *K*-mer analysis, we predicted the genome size of ‘Wanfeng’ almond to be 288.08 Mb, with a heterozygosity rate of 0.81% and a repeat rate of 50.98%. Using the PacBio Sequel II sequencing platform, we obtained 27.57 Gb of HiFi data for ‘Wanfeng’ almond genome assembly (Table 1). The assembled genome size was 288.53 Mb, which is close to the predicted size from the genome survey analysis. The contig N50 and scaffold N50 were 30.48 Mb and 31.36 Mb, respectively. Additionally, using Hi-C technology, we anchored the ‘Wanfeng’ almond genome to 8 chromosomes. The base statistics of the ‘Wanfeng’ almond genome showed that the GC content was 112,817,304 bp (39.1%), the AT% content was 175,710,174 bp (60.89%), and the N content was only 2,000 bp (Table 6). We re-compared the DNB-seq (DNBSEQ sequencing technology.) and HiFi data to the ‘Wanfeng’ almond genome, which showed coverage above 96% (Table 7). The genome completeness was assessed by BUSCO analysis, with 1599 complete BUSCO genes detected (99.3%). These data results indicate the completeness and accuracy of the genome assembly of ‘Wanfeng’ almond, and provide reference genomic background information for subsequent molecular breeding of almond in China.

**Table 6:** Base composition statistics of ‘Wanfeng’ almond genome.

Types	Length (bp)	% of Genome
A	87,898,644	30.46
T	87,811,530	30.43
C	56,523,771	19.59
G	56,293,533	19.51
N	2,000	0
GC	112,817,304	39.1
total	288,529,478	100

**Table 7:** Comparison of ‘Wanfeng’ almond genome.

Types	Mapping rate (%)	Average sequencing depth	Coverage (%)	Coverage (> = 5X, %)	Coverage (> = 10X, %)	Coverage (> = 20X, %)
DNB-seq	96.53	148.01	99.99	99.98	99.95	99.86
HiFi	100	83.21	99.41	98.51	98.14	97.38

## Data Availability

This study did not involve code application.
